# Projecting 1 km-grid population distributions from 2020 to 2100 globally under shared socioeconomic pathways

**DOI:** 10.1038/s41597-022-01675-x

**Published:** 2022-09-12

**Authors:** Xinyu Wang, Xiangfeng Meng, Ying Long

**Affiliations:** 1 School of Architecture, Tsinghua University, Beijing, 100084 China; 2 School of Architecture and Hang Lung Center for Real Estate, Key Laboratory of Eco Planning & Green Building, Ministry of Education, Tsinghua University, Beijing, 100084 China

**Keywords:** Socioeconomic scenarios, Decision making, Social sciences, Geography, Energy modelling

## Abstract

Spatially explicit population grid can play an important role in climate change, resource management, sustainable development and other fields. Several gridded datasets already exist, but global data, especially high-resolution data on future populations are largely lacking. Based on the WorldPop dataset, we present a global gridded population dataset covering 248 countries or areas at 30 arc-seconds (approximately 1 km) spatial resolution with 5-year intervals for the period 2020–2100 by implementing Random Forest (RF) algorithm. Our dataset is quantitatively consistent with the Shared Socioeconomic Pathways’ (SSPs) national population. The spatially explicit population dataset we predicted in this research is validated by comparing it with the WorldPop dataset both at the sub-national and grid level. 3569 provinces (almost all provinces on the globe) and more than 480 thousand grids are taken into verification, and the results show that our dataset can serve as an input for predictive research in various fields.

## Abstract (summary)

**Table:** 

Measurement(s)	Global future grid population distribution from 2020 to 2100
Technology Type(s)	Random Forest algorithm
Factor Type(s)	Global projections of future wilderness in 2100 • Travel time to cities in 2015 • WorldPop in 2015 and 2020 (unconstrained global population grids) • Global Urban Land Use Change Product (GULCP, under 5 SSPs) from 2020 to 2100 (10-year intervals) and in 2015 • Global DEM in 2012 • Global Roads • Global Land Cover in 2015 • SSPs population projections from 2020 to 2100

## Background & Summary

Global climate change and sustainable development are receiving increasing attention both from researchers and policymakers^1^. Human activities have contributed to major greenhouse gas emissions and resource consumption at both regional and global levels^2^. Global spatially explicit gridded population data is the key to achieving the two-carbon goals (carbon neutrality and peak carbon dioxide emissions) and SDGs. In addition, it can support studies in multiple fields, such as economic development, resource management, and urban and rural development^3–6^. Therefore, research on the future trends of global population distribution is a worthy topic for scientists to further explore^7,8^.

Since the 1990s, there have been growing attempts to decompose national level population datasets into regular spatially distributed grids^9^. Tobler *et al*.^10^ produced the earliest spatially explicit population grid for the globe with a resolution at 5 arc-minutes in 1997, and this work has been continuously updated by the Center for International Earth Science Information Network (CIESIN) ever since. The latest version of it, GPWv4, was released in 2015^11^. The global spatially explicit population grids generated by various methods also include LandScan^12^, WorldPop^5^, GHS-POP^13^, and WPE^14^, which have different time spans and spatial resolutions (Table 1).

**Table 1:** Existing datasets of gridded population of the globe.

Dataset		Time Span	Resolution	Sources
Historical population data on the globe	Gridded population of world (GPWv4)	2000, 2005, 2010, 2015, 2020	30 arc-seconds	https://sedac.ciesin.columbia.edu/data/collection/gpw-v4/
	Global Rural-Urban Mapping Project (GRUMPv1)	1990, 1995, 2000	30 arc-seconds	https://sedac.ciesin.columbia.edu/data/collection/grump-v1
	LandScan	2000–2019	30 arc-seconds	https://www.eastview.com/resources/e-collections/landscan/
	WorldPop	2000–2020	30 arc-seconds	https://www.worldpop.org/
	Global Human Settlement Layer-Population (GHS-POP)	1975, 1990, 2000, 2015	250 m/1 km/9 arc-seconds/ 30 arc-seconds	https://ghsl.jrc.ec.europa.eu/ghs_pop2019.php
	World Population Estimate (WPE)	2013, 2015, 2016	150 m	https://www.arcgis.com/home/item.html?id=92d3005feb84428a8f85160f2451ec63
Projection population data for the globe	Related gridded population projection datasets on the globe	1990–2100	0.5 arc-degree	Bengtsson *et al*.^2^
		2000–2100	7.5 arc-minutes	Jones *et al*.^15^
		2000–2100	1 km	Gao^16^
		1980–2100	0.5 arc-degree	Murakami *et al*.^17^
Projection population data for regions	Spatially explicit projection of US population	2030, 2050	30 arc-seconds	McKee *et al*.^20^
	High-resolution African population projections	2000–2100	30 arc-seconds	Boke-Olén *et al*.^21^
	Provincial and gridded population projection for China	2010–2100	30 arc-seconds	Chen *et al*.^22^
	High-resolution gridded population projections for China	2015–2050	100 m	Chen *et al*.^23^

Since the 2000s, with the rapid development of economic globalization and urbanization, the demand for spatially explicit population projection is gradually increasing, and some projection datasets have also been produced (Table 1)^2,15–17^. The main methods utilized for global population projection can be summarized as two steps: calculate population potential surfaces (or dasymetric weighting layer) and allocate administrative level population to grid level. Bengtsson *et al*.^2^ presented a dataset covering global population forecasts for the period of 1990–2100 at a 0.5-degree resolution. What’s more, this study also presented a gridded dataset of urban and rural populations for the period of 1990–2050 under the IPCC Special Report on Emission Scenarios (SRES)^18^. This work aggregated the 1 km LandScan dataset to 0.5-degree for producing potential surface and calibrated population grid with country projections. Jones *et al*.^15^ based on the 2.5 arc-minute Gridded Population of the World (GPW) in 2000 and utilized a parameterized gravity-based downscaling model to calculate population potential surfaces, and predicted the global spatial population (2000–2100) with Shared Socioeconomic Pathways (SSPs)^19^. Moreover, Gao^16^. downscaled this work^15^ into 1 km by using Global Rural-Urban Mapping Project version 1 (GRUMPv1) in 2000 as a potential surface and obtained a new population projection for 2000–2100 under five SSPs. Although Gao’s work has a fine resolution, it is a significant short disadvantage that the accuracy and continuity of this work are not suitable for fine spatial resolution research (e.g., urban and rural development research need to know the population distribution in urban areas, which means the kilometer-level grid is needed) because the input data are not designed to offer these demands. Murakami *et al*.^17^ used a series of models considering road density, urban population, and distance to airport/ocean for producing population potential surface. Based on these surfaces, this work produced 0.5 arc-degree resolution global gridded population dataset by downscaling urban and non-urban SSPs populations from 2010 to 2100. These works make valuable exploration in producing spatially explicit population grid, but these global population distribution datasets either not sufficient for fine spatial resolution applications or are out of date.

Since 2010, the resolution of spatially explicit population projections has made great progress. Some researchers have developed new methodologies and produced high-resolution spatially explicit projections at the regional level, such as in the United States (US)^20^, Africa^21^, and China^22,23^, rather than at the global level. Above all, Chen *et al*.^23^ applied three machine learning algorithms to 100 m resolution population grid predictions in China from 2015 to 2050 and achieved excellent accuracy, confirming the method’s effectiveness in generating global high-resolution population grid potential applications.

Here, we follow the machine learning method^22^ and present an approximately 1 km (30 arc-seconds) global projection under five SSP scenarios for 2020–2100 at 5-year intervals, based on the open-access WorldPop dataset^5^. WorldPop is a high-resolution population dataset that is a key component of many studies. WorldPop has been used in resource allocation, disaster management, transport and city planning and environmental impact assessment (https://www.worldpop.org/about/), including for example, estimating the impact of the 2015 Nepal earthquakes^24,25^, guiding medical resource allocation of India^26^, assessing global rural accessibility and rural roads investment^27^. Our dataset is designed for fine resolution research and has a broad application prospect for climate change, urban development, public health research and other fields.

## Methods

**Fig. 1:**
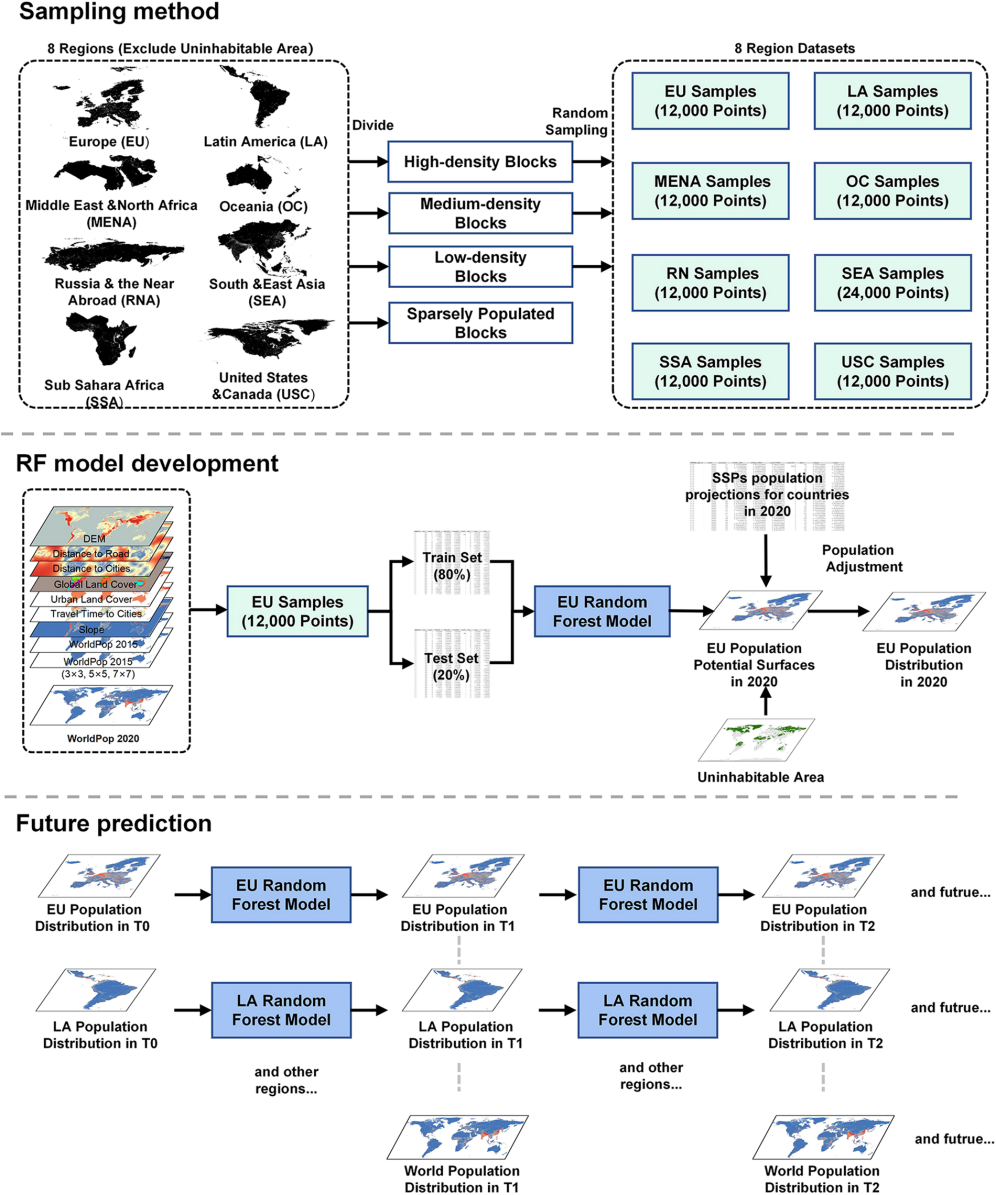
Flow chart of the methodology of this research. Our research includes three procedures: sampling method, random forest (RF) model development and future projection. In procedure one (Sampling method), we exclude uninhabitable areas and divide the world into 8 regions for model training, i.e., Europe (EU), Latin America (LA), Middle East & North Africa (MENA), Russia & the Near Abroad (RNA), Sub-Sahara Africa (SSA), United States & Canada (USC), Oceania (OC) and South & East Asia (SEA) and sample enough points randomly from each region. In procedure two (RF model development), Europe (EU) is taken as an example. Multiple input datasets are extracted as a table based on the EU samples. These values are divided as train and test sets for the EU RF model, and the trained model is utilized to produce EU population potential surfaces. SSPs are used as a total population constraint at the national level. In procedure three (Future projection), we conduct cyclical projections according to time series (5-year intervals) for EU. Furthermore, all 8 regions are predicted as in procedure two. Finally, we merge results to obtain the final population projections for the globe.

We predict the global spatially explicit population grid from 2020 to 2100 by building an RF model based on spatial path dependence. The spatial path dependence^28^ can reflect the influence of initial or early conditions on process evolution, and suppose the population distribution at time T2 is affected by the distribution at time T1 as well as other environmental factors. Based on methods^17,22,23^ utilized for population projection (calculate population potential surfaces and allocate administrative level population), our process utilized a random forest algorithm for calculating population potential surfaces because of the excellent predictive performance and wide application in population prediction^23,29^. Our method involves three procedures: (1) preparations before projection: considering that there are large disparities between different regions on the globe, dividing countries/territories into 8 regions^30^, and randomly sampling enough points in these regions to develop our RF model based on 2015 WorldPop (see section **Sampling method**). (2) calculating projection model: training apposite model for each region and calculating population potential surfaces (see section **RF model training**) and (3) conducting future projection at 5-year intervals for each region under five SSPs (see section **Future prediction**). The method framework of this research is shown in Fig. 1 and details of each procedure will be explained below.

### WorldPop dataset

The WorldPop project^31^ provides global gridded population data at a resolution of 30 arc-seconds (~1 km at the equator). WorldPop’s strength is that its model is able to identify significant relationships from incoming census data and ignore rural areas without obvious satellite-derived built-up areas^32^. WorldPop also makes all source code publicly available and method transparent, and integrates various inputs and auxiliary data so that models can use different weights to redistribute populations between census or administrative unit counts^33^. One of the major weaknesses and criticisms of “WorldPop” is that its model has no other constraints except for water bodies, and the dataset dasymetrically redistribute population in administrative units throughout the whole unit areas, not just within the grid cells classified as “built-up”.

Based on the strengths and weaknesses of the WorldPop dataset, combined with the comparative analysis results of the released global gridded population datasets (including GPW, GHS-POP, WorldPop, and LandScan) by Yin *et al*.^34^, and considering the problem of data time series, we decided to use the unconstrained global population grids as the population input data for this study.

### Other source datasets

The existing studies have shown that the spatial distribution of population is affected by comprehensive factors such as economy, policy, environment, and resources^20,23^. Therefore, considering the availability of data, several environmental factors widely used in existing research^20,21,35–38^ were taken as input datasets to conduct our spatial projection, including travel time to cities^38^ DEM, slope, distance to road, distance to cities, Global Land Cover (mainly focuses on natural conditions)^39^ and Global Urban Land Use Change Product (GULCP), the world’s first 1-km resolution maps of future global urban land predicted under the SSP framework using the FLUS model. The high-resolution GULCP preserves spatial details and can avoid the distortions in global urban land patterns^40^. Significant differences in the predicted paths of future urban development among the five scenarios are that Scenario SSP5 has an increasing trend and the largest urban land area, scenario SSP2 and SSP3 produce similar trends to SSP5, but with much smaller urban land areas. For the SSP1 and SSP4 scenarios, the urban land demand is expected to decline in the 2080s and 2070s, respectively, due to a hypothetical slowdown in socioeconomic growth^40^. The projections are comparable to three existing representative global urban land projections by Chen *et al*.^40^, and the results show that GULCP has high resolution and is precise, which can enhance support the research in other related disciplines, such as ecological protection, urban climate and global climate change. Furthermore, the surrounding population distributions of each grid were also taken into consideration based on existing researches^23,40^. The source datasets used for the global spatially explicit population projection are listed in Table 2. The input raster layers are listed in Supplementary Table 1.

**Table 2:** Source datasets used for the global gridded population projection.

Name	Resolution	Temporal domain	Type	Source
Global Administrative Boundaries	—	2018	Polygon	Natural Earth Data (https://www.naturalearthdata.com/downloads/)
Global projections of future wilderness	1 km	2100	Raster	Li *et al*.^47^
Travel time to cities	~1 km (30 arc-seconds)	2015	Raster	Weiss *et al*.^38^
WorldPop (unconstrained global population grids)	~1 km (30 arc-seconds)	2015, 2020	Raster	https://www.worldpop.org/
Global Urban Land Use Change Product (GULCP, under 5 SSPs)	1 km	2015, 2020-2100	Raster	Chen *et al*.^40^
Global DEM	200 m	2012	Raster	https://www.nasa.gov/topics/earth/index.html
Global Roads	—	—	Polyline	Global Roads Open Access Data Set, Version 1 (gROADSv1): http://sedac.ciesin.columbia.edu/data/set/groads-global-roads-open-access-v1
Global Land Cover	5 km	2015	Raster	Dynamics of Global Land Cover (http://data.ess.tsinghua.edu.cn/)
SSPs population projections	Country-level	2020–2100	Text	SSP Database (https://tntcat.iiasa.ac.at/SspDb/)

### Shared socioeconomic pathways scenario (SSPs)

The SSPs used in this study are a set of future pathways of societal development that are developed for use in global climate change research^3,41^. The SSPs describe five alternative outcomes of trends in demographics, economic development, urbanization and so on that are provided by the International Institute for Applied Systems Analysis (IIASA)^41,42^. The five population scenarios are colloquially named SSP1 (Sustainability), SSP2 (Middle of the Road), SSP3 (Regional Rivalry), SSP4 (Inequality), and SSP5 (Fossil-fuelled Development) (Table 3)^21^. This study follows the population projection data made by IIASA^42^ and urban land expansion projections made by Chen *et al*.^40^ to simulate future population changes for the globe. The SSP dataset and more research on the SSPs can be found at the following link: https://iiasa.ac.at/web/home/research/researchPrograms/Energy/SSP_Scenario_Database.html.

**Table 3:** Population fertility, mortality and migration under different SSPs^21^.

SSPs	SSP1	SSP2	SSP3	SSP4	SSP5
	Sustainability	Middle of the Road	Regional Rivalry	Inequality	Fossil-fueled development
Fertility	Low	Medium	High	Low	Low
Mortality	Low	Medium	High	Medium	Low
Migration	Medium	Medium	Low	Medium	High

### Sampling method

Due to the huge number of pixels of the population grid, sampling across sub-regions is urgently needed before predicting. There is less related research on how to sample population grids scientifically, so we tried some sampling methods, such as random, cluster, systematic, and stratified random sampling^43^, to explore which sampling method was more suitable for this work. The experimental results proved that population distribution on the globe is extremely uneven, so a large number of noise grids (sparsely populated grids) will be obtained by systematic and random sampling. This will reduce the interpretation of RF model. Cluster sampling will select all grids being concentrated in a certain area, which is not conducive to prediction for the globe. Chen *et al*.^23^ raised a stratified random sampling method by dividing explicit population grids into four kinds of 250 km blocks (i.e., high-density, medium-density, low-density, and sparsely populated), and collecting sample points in the first three kinds^23^. They equally allocated 2,000 points from each block for machine learning model building and obtained reliable projection data. Although the sample placement (the distribution of 250 km blocks) may have more effect on accuracy than the sampling method^23^, the representativeness of each block was enhanced by considering whether there were significant cities within the block.

**Fig. 2:**
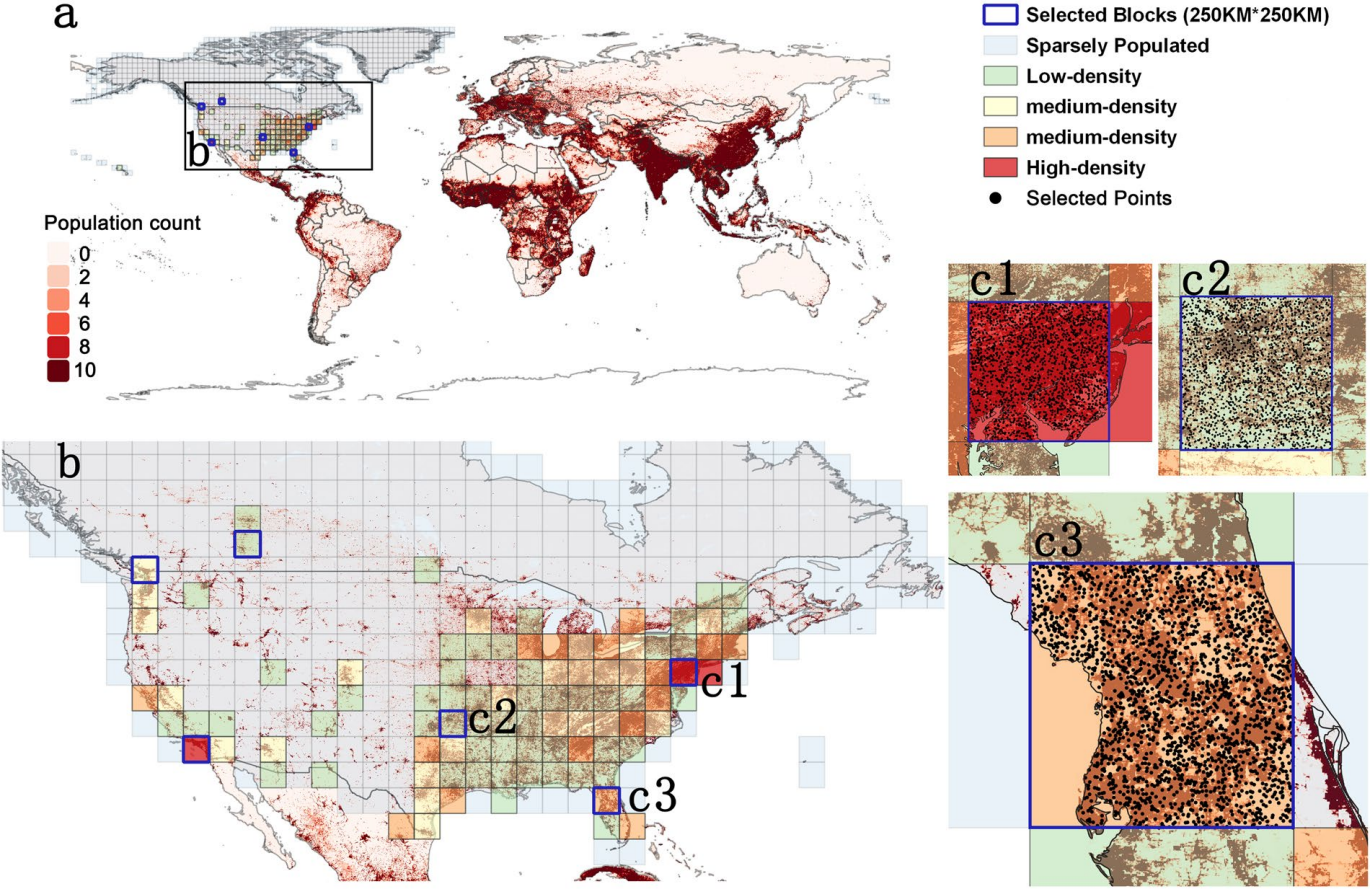
Sampling method (the United States & Canada, USC is taken as an example). (**a**) shows the distribution of USC. The gradient from white to red in the bottom graph shows the world population distribution in 2015 (from WorldPop). (**b**) shows the distributions of high-density, medium-density, low-density, and sparsely populated blocks, and blocks with blue borders (including c1, c2 and c3) are selected for subsequent sampling. (c1, c2 and c3) show the distribution of sample points (each block contains 2000 points). All the points are randomly distributed on the land, not the ocean. The minimum distance between any two points is greater than 1.5 km, which means they are not in the same 1 km grid.

**Fig. 3:**
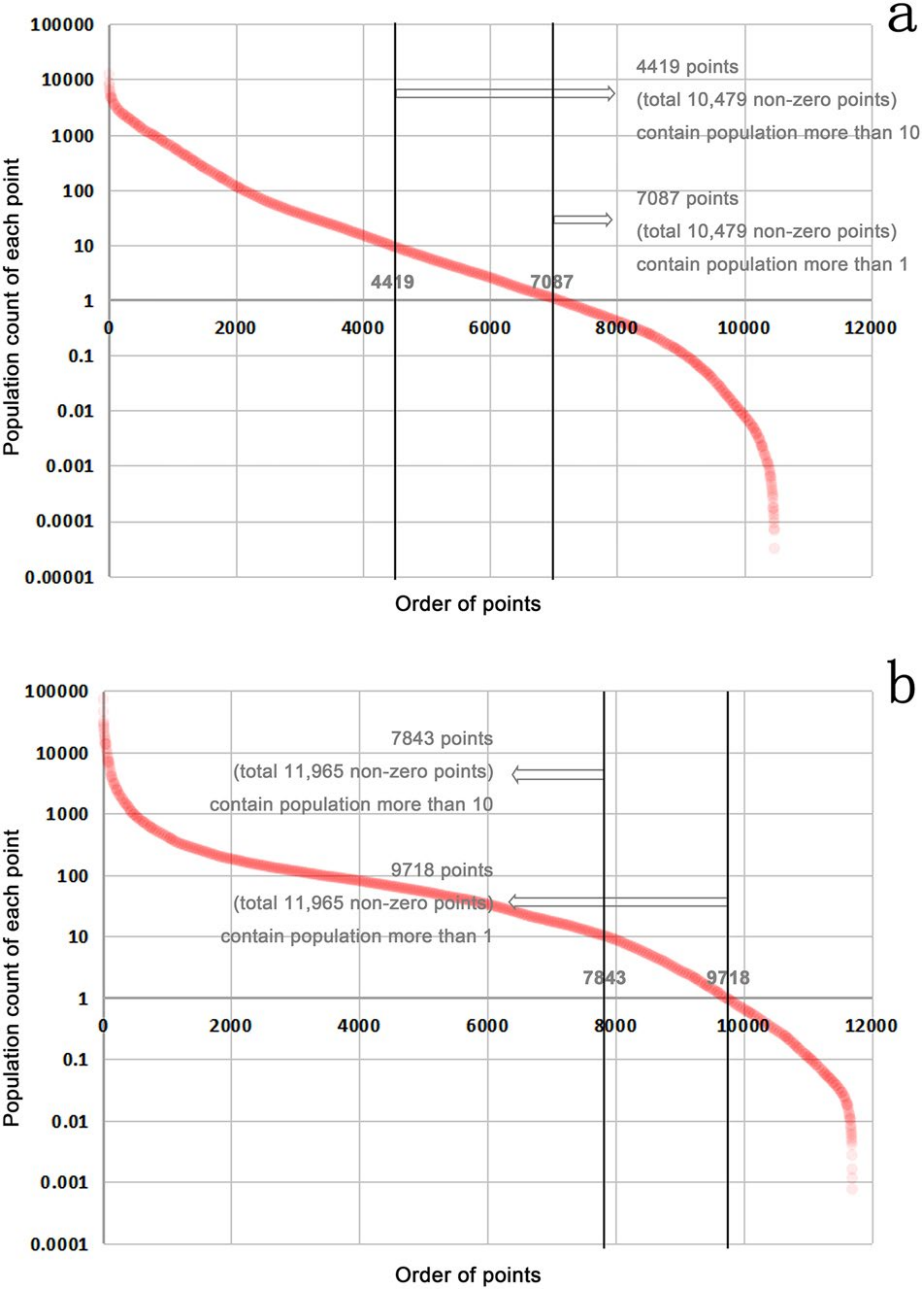
Distribution of sampling points. The x-axis represents the points in the order of population from maximum to minimum, and the y-axis (logarithmic axis) is the estimated value of the population count of each point, which may be a decimal. Each red point represents one sampling point. (**a**) shows the 12000 sampling points in the **United States & Canada (USC)** from 6 blocks. Of the 10479 non-zero points, 4419 points (42.7%) have a population of more than 10, and 7087 points (67.6%) have more than 1. (**b**) demonstrates the 12000 sampling points in **Sub-Sahara Africa (SSA)** from 6 blocks. Of the 11965 non-zero points, 7843 points (65.5%) have a population of more than 10, and 9718 points (81.2%) have more than 1.

The specific descriptions of this sampling method are as follows (see Fig. 2). First, we tessellate the territory of 8 regions by 250 km blocks and calculate the population density of each block. Second, we divide each region into more than 4 types and select enough 250 km blocks for 8 regions, ensure that there is at least one important city (capital, provincial capital or economic center) inside each block and consider its spatial location (try to make blocks evenly distributed in each position, rather than clustering in a certain area). Then, we select 6 blocks for each region (2 high-density, 2 medium-density and 2low-density blocks)^23^. However, due to the massive population of SEA (more than 3 billion in 2020) and the small population of OC (about 30 million in 2020), we adjust the number of blocks and select 3 in OC (1 block for each) and 12 blocks in SEA (4 blocks for each), respectively. Third, 2000 points are sampled randomly in each block for building RF model. The third step has strong robustness as shown in the validation part (see Supplementary Table 2). To reduce the risk of oversampling from lightly populated areas, we conduct statistical analysis, and Fig. 3 demonstrates that these sampling points are reliable. Finally, we utilize 8 region datasets to build our RF model.

### RF model development

We build RF models for 8 regions respectively, and EU is taken as an example (Fig. 1). Based on the 12,000 EU sample points, values of all input datasets are extracted as a table. These values are divided as train set (80%) and test set (20%) for EU RF model training. We train each model 20 times and select the most accurate one for producing EU population potential surfaces. The performance of each RF model is verified. We exclude the uninhabitable areas and take SSPs as the total constraint at the national level. Moreover, Urban Land Use dataset produced by Chen *et al*.^40^, which predicts the future urban expansion (2020–2100) under five SSP scenarios, is also used as input data, and they will change as the year goes (5-year intervals), which can help to better simulate the development of future population distribution.

### Future projection

In this procedure, we conduct cyclical projections according to time series (5-year intervals) for all regions. Population distribution (WorldPop dataset), SSPs population projections at the country level and Urban Land Use dataset are changing over time as input datasets for simulating SSPs. Finally, we merge 8 regions’ population projection results to obtain the final projection dataset for the globe.

However, the population data provided by the SSPs (188 countries or areas in this research) does not cover every country and area on the globe. For those 60 countries or areas without SSPs projection data, we skip the population adjustment step. And the final population dataset we predicted covers 248 countries or areas on the globe. The list of countries is shown in Supplementary Table 3.

**Fig. 4:**
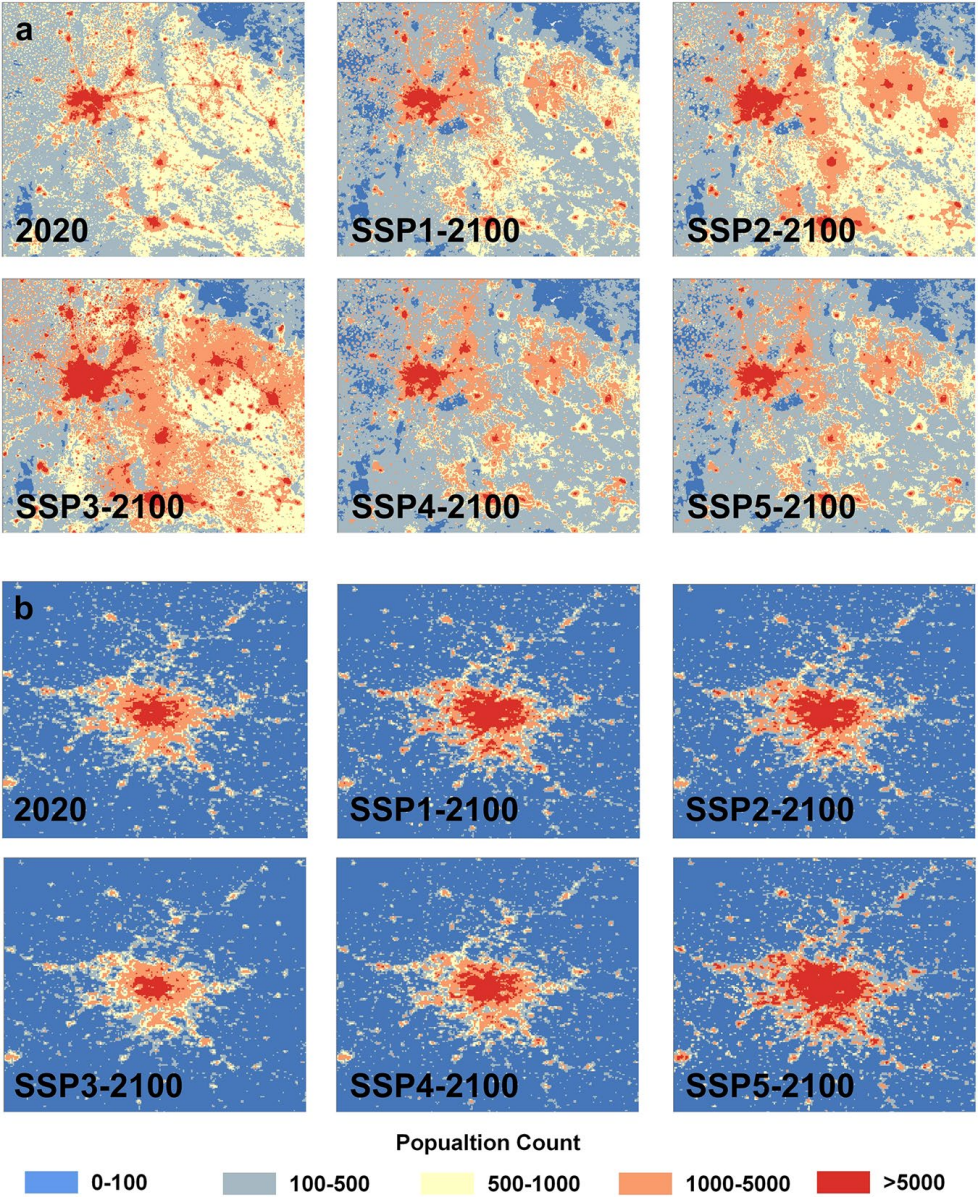
Two examples are selected to show the population distributions for 5-SSP scenarios in 2100 and 2020. (**a**) is New Delhi and surrounding cities in India. (**b**) is Paris in France.

Finally, we compare the differences between five SSPs by selecting two examples on the globe in 2100, as shown in Fig. 4. It can be seen from the figure that the population distribution under the five scenarios is substantially different. The future development of population is complex, which is the result of the intersection of the country’s total population and urbanization development pattern. Under the SSP3 scenario, Paris’ population may shrink in 2100 compared with 2020 because of the decrease of France’s population, but the population of New Delhi and surrounding cities may increase. The population of India in 2100 is essentially the same under the SSP1 and SSP5 scenarios. Under SSP5, the urban area of New Delhi and surrounding cities may expand more widely than under SSP1. For the area close to cities, this could lead to an increase in population. But for further areas, the population may decrease. Government, organizations, or researchers can utilize this dataset in different scenarios according to their research objectives, such as sustainable development, global climate change, energy consumption and so on.

## Data Records

The projected gridded global population data under five SSP scenarios from 2020 to 2100 are stored as a GeoTIFF file (.tif) with the WGS84 projection at approximately 1 km (30 arc-seconds) resolution. These can be freely and publicly accessed at Figshare. The dataset can be downloaded at Figshare (10.6084/m9.figshare.19608594.v2)^44^.

## Technical Validation

The technical validation of our dataset is performed in four parts: (1) robustness test for sampling method, (2) performance of RF model on test sets, (3) comparison of predicted values and observed values, and (4) comparison of our dataset with published related datasets. Considering input datasets, the third comparison can be only verified in 2020, whereas the last can be verified in both 2020 and the future.

We use MAE (Mean Absolute Error), which reflects the overall accuracy of the projections, and RMSE (Root-Mean-Square-Error), which reflects the bias of the projections, and %RMSE, which eliminates the influence of population size on RMSE, to verify our projection at the sub-national level. These metrics are commonly used to evaluate the accuracy of population projections. The equations for the indicators are as follows, where *y*_*i,pre*_, *y*_*i,obs*_ represent the predicted and observed value for grid *i*, respectively. *n* is the number of grids. ${\overline{y}}_{i,obs}$ represents the mean value of the observed dataset.

1 $$MAE({y}_{pre},{y}_{obs})=\mathop{\sum }\limits_{i=1}^{n}\frac{| {y}_{i,pre}-{y}_{i,obs}| }{n}$$

2 $$RMSE({y}_{pre},{y}_{obs})=\sqrt{\mathop{\sum }\limits_{i=1}^{n}\frac{{({y}_{i,pre}-{y}_{i,obs})}^{2}}{n}}$$

3 $${\rm{ \% }}RMSE({y}_{pre},{y}_{obs})=\frac{RMSE({y}_{pre},{y}_{obs})}{{\overline{y}}_{obs}}\times 100{\rm{ \% }}$$

Keeping the 250 km grids unchanged, we conduct the sampling method 20 times for EU and other 7 regions, and calculate MAE, and RMSE by comparing the predicted values with observed values for each model (As shown in Table 4 for EU and Supplementary Table 2 for all 8 regions). The results are stable, which shows our sampling method is robust.

**Table 4:** Robustness test for sampling method.

Model	MAE	RMSE	Model	MAE	RMSE
Sample1	10.07	125.90	Sample11	9.91	103.54
Sample2	10.19	111.63	Sample12	10.55	131.89
Sample3	10.44	123.42	Sample13	10.42	128.82
Sample4	9.83	107.60	Sample14	10.20	121.09
Sample5	9.95	129.40	Sample15	9.83	135.39
Sample6	10.47	121.69	Sample16	10.21	140.97
Sample7	9.90	110.11	Sample17	10.20	154.45
Sample8	10.58	123.24	Sample18	9.58	88.65
Sample9	10.48	131.16	Sample19	10.72	135.63
Sample10	10.20	135.49	Sample20	10.57	140.37

This table shows the Robustness test results for EU.

Table 5 shows the performances of 8 RF models’ test sets. The Number of Trees (a hyperparameter of RF model) for 8 models is 500, which is the same as the existing studies^23^. The %RMSE of our models ranges from 7.65% to 47.85%, the same level as results made by Chen *et al*.^23^ for China (7.78%–24.84%).

**Table 5:** Performances of 8 RF models on their test sets.

Name	No. of Training Samples	MAE	RMSE	%RMSE
Europe (EU)	9,600 (12,000 × 0.8)	9.57	35.20	17.94
Latin America (LA)	9,600 (12,000 × 0.8)	18.39	106.36	41.37
Middle East & North Africa (MENA)	9,600 (12,000 × 0.8)	36.11	189.54	47.72
Oceania (OC)	4,800 (6,000 × 0.8)	2.59	11.27	14.87
Russia & the Near Abroad (RNA)	9,600 (12,000 × 0.8)	5.38	37.27	47.85
South & East Asia (SEA)	19,200 (24,000 × 0.8)	28.19	122.2	19.12
Sub-Sahara Africa (SSA)	9,600 (12,000 × 0.8)	9.10	43.16	19.05
United States & Canada (USC)	9,600 (12,000 × 0.8)	3.60	16.20	7.65

Number of Trees (a hyperparameter of RF model) for all 8 models are 500. Comparison of predicted values and observed values.

Before validation, we first adjust our dataset in 2020. As shown in **RF model development**, we take SSPs population projections as the total constraint at the national level, but the observed values are under assumption made by WorldPop dataset, not the SSPs. To eliminate the influence of technical validation caused by this difference, we adjust our dataset according to the national population aggregated from WorldPop 2020, and regard this as predicted values for further validation.

**Fig. 5:**
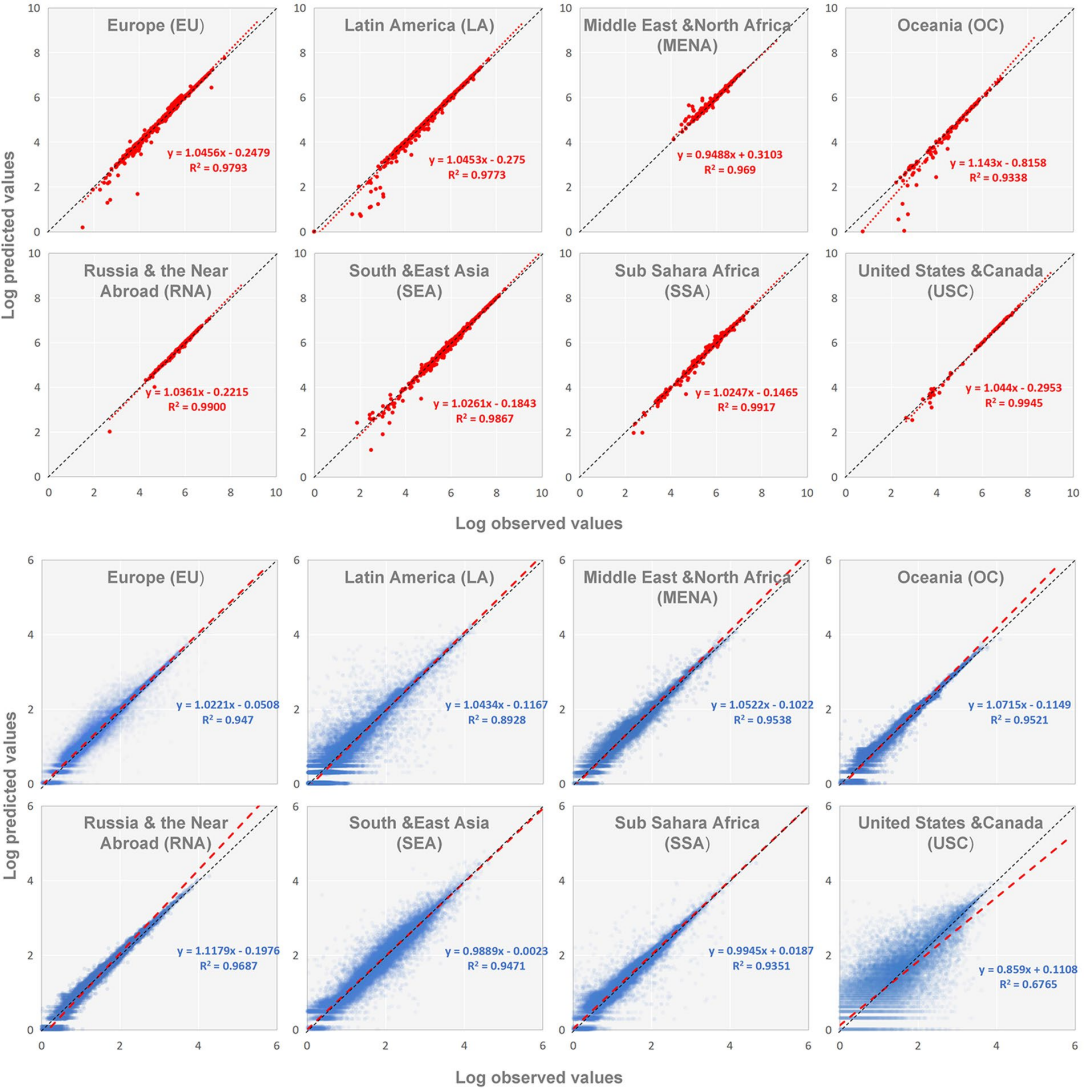
Comparing the observed and predicted values at the sub-national and grid level on the globe in 2020. The top 8 figures (red points) show the result of the sub-national level and the others (blue points) show the result of the grid level. The values of MAE, RMSE and %RMSE in each figure are shown in Table 6.

We conduct validation both on the sub-national and grid level. For the sub-national level, grid population values are aggregated by provincial boundaries from GDAM (as shown in Fig. 5, each red point represents one province). For grid level, we sample 100,000 points randomly in each region (including numerous sparsely populated points). Points with a population of less than 1 are eliminated, and we make sure each region has more than 50,000 points who participated in the verification (as shown in Fig. 5, each blue point represents one population point randomly selected from each region). Table 6 shows the projection errors both at the sub-national and grid level by comparing predicted and observed values (WorldPop 2020), and the distributions of these values are shown in Fig. 5. The %RMSE values of 8 regions are ranged from 5.51% to 59.73% (Table 6, sub-national level), which are acceptable compared to the results of Sorichetta *et al*.^37^ (%RMSE values are 52.96%–259.81% for LA sub-national administrations population projection). Compared with the validation results from Boke-Olén *et al*.^21^ (RMSEs are 26,917–1,162,510 for SSA sub-national administrations), our validation results show that our population projection results are accurate (RMSE is 313,968.62 for SSA). The MAE of our dataset for SEA at grid level is 55.57 (Table 6, ~1 km grid level), which is nearly equal to the validation results of Chen *et al*.^22^ (49.7–58.2). All these comparisons demonstrate that our predictive method and global gridded population projection products are reliable, which can provide support for research in other fields.

**Table 6:** Evaluation of overall errors of global population projections at the sub-national and grid level in 2020.

Region	Spatial domain	n	MAE	RMSE	%RMSE
EU	Sub-national	711	52730.63	478416.31	59.73
LA	Sub-national	652	48060.45	190091.32	17.90
MENA	Sub-national	400	75126.49	150158.48	10.13
OC	Sub-national	122	14060.64	29714.38	9.70
RNA	Sub-national	235	40898.01	68102.21	5.51
SEA	Sub-national	679	231773.23	641521.20	10.24
SSA	Sub-national	689	97934.58	313968.62	19.35
USC	Sub-national	81	157345.00	390167.78	8.33
EU	~1 km Grid	64669	14.49	123.33	143.35
LA	~1 km Grid	70010	19.81	184.72	247.26
MENA	~1 km Grid	50345	11.86	113.38	176.35
OC	~1 km Grid	54234	3.76	29.92	66.57
RNA	~1 km Grid	59326	4.29	33.16	89.05
SEA	~1 km Grid	70430	55.57	369.71	184.19
SSA	~1 km Grid	69144	10.85	137.25	224.23
USC	~1 km Grid	51239	43.51	179.02	212.46

For the sub-national level, we verify all sub-national administrations, except those with sparely population. For grid level, we sample and verify enough points (more than 50,000) randomly for each region.

### Comparison with other datasets

**Fig. 6:**
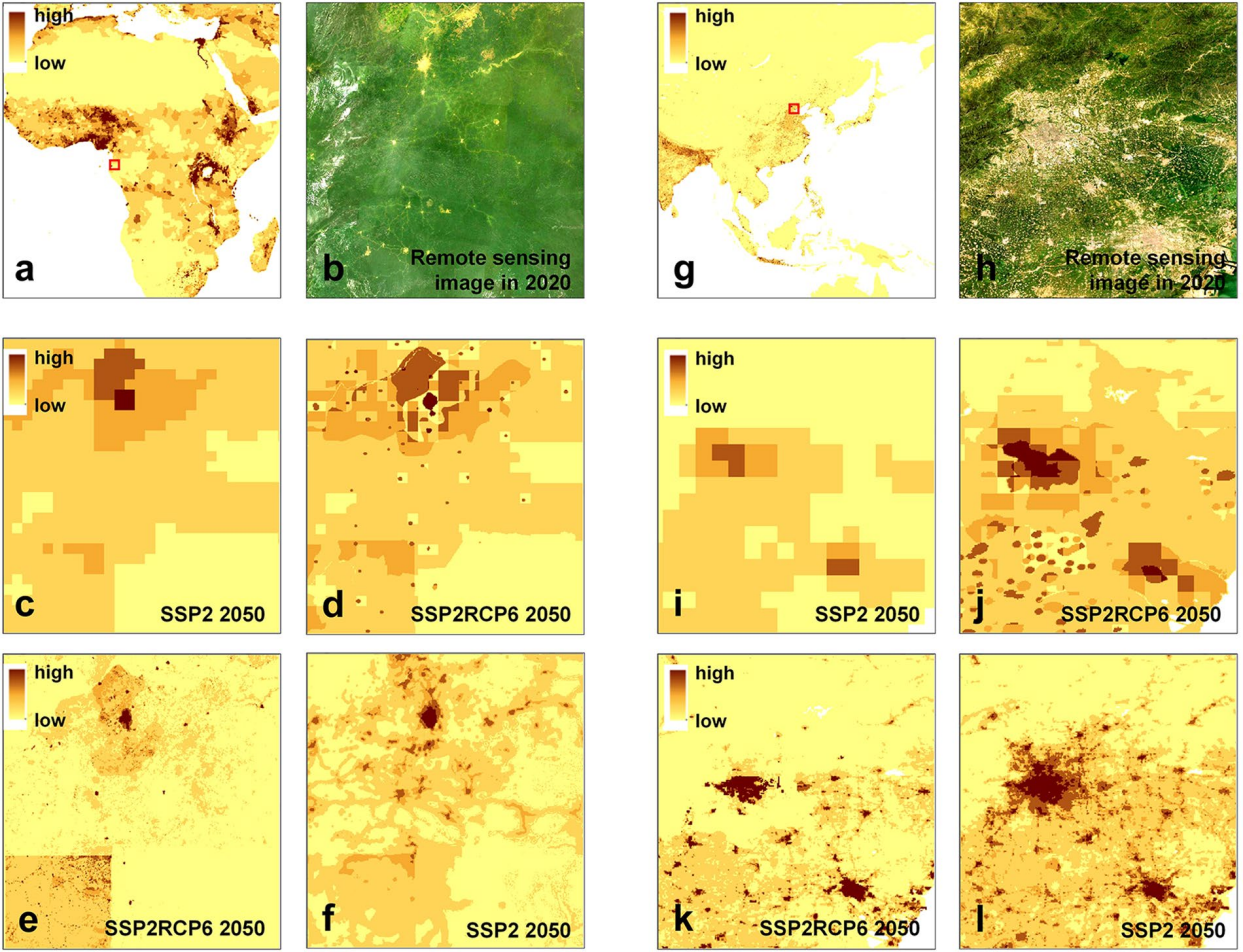
Comparison with existing related datasets: two sample regions. Figures **a & g** are the location of two sample regions. Figures **b & h** are remote sensing images in 2020 from ESRI images. Figures **c & i** (7.5 arc-minutes grid) are population projection data of the globe under SSP2 in 2050 from Jones & O’Neill. Figures **d & j** (1-km grid) are prediction data of the globe under SSP2 in 2050 from Gao. Figure **e** (~1-km grid) is projection data of Africa under SSP2RCP 6 in 2050 from Boke-Olén *et al*. Figure **k** (~1-km grid) is projection data of China under SSP2RCP6 in 2050 from Chen *et al*. Figures **f & l** (~1-km grid) are projection data of the globe under SSP2 in 2050 of this research. Colours show relative highs and lows within each map. The resolution of Figures **d, e, f, j, k** and **l** are 1 km or ~ 1 km, and their legends are unified. The resolution of Figures **c & i** are 7.5 arc-minutes, and their legends are the same, but different from the former.

Existing related datasets, including projection datasets for the globe^15,16^ and regions^21,22^, are taken into comparison. Figure 6 shows that our dataset seems to better fit with the current remote sensing image compared to the other datasets and smoother compared to the city level datasets in Africa and China. This means that our dataset offers the possibility to compare population development patterns at the city scale under different SSP scenarios. We have made a preliminary discussion in the **Future projection** part.

### Strengths, limitations and uncertainties

The first strength of this dataset is the application of machine learning methods, which can identify the vital relationship between different input datasets. The second strength is the continuous time series. This dataset is designed for comparing over time. The third strength is adaptability to other studies. Some input datasets (GULCP and SSPs population projection at the national scale) are changing from year to year, which means our projections are consistent with these studies. The fourth strength is that this population projection matches with satellite better than other related studies, which means this dataset can be applied to the differences in development population patterns under 5 SSP scenarios.

However, our study still has some limitations. First, although this dataset is capable of demonstrating different population patterns among 5 SSP scenarios for the same city, it fails to consider the urbanization rate. It means that this dataset and other urban land cover datasets (e.g., GUCLP) should not be combinedly used for calculating urbanization rates. Second, WorldPop population in 2015 and 2020 may be based on the same underlying input population data^45^ which may cause the validation results (especially the result in Fig. 5 and Table 6) to appear better than they are.

Moreover, there are still method and policy uncertainties in this study that may affect the predicted results. For method uncertainties, the interval of GULCP is 10 years, but our projection data is 5 years. We had to use GULCP 2020 instead of GULCP 2025 as the urban land use input data to predict population distribution in 2025; Second, the RF model of USC has a low %RMSE value on the test set (Table 5) but the overall projection result is not ideal (Fig. 5), indicate that the model may be affected by noisy data or the samples are not well represented, which requires further research. However other regions’ model does not occur this error.

For policy uncertainties, China has implemented population ceiling policies in mega-cities, so the population growth of them may be limited. The model for this study does not consider the impact of policy factors on population distribution. In addition, due to ethnic, energy, and territorial issues, some countries such as Afghanistan, Israel, and Iraq are affected by war year-round, and their population changes lack regularity. Moreover, diseases, natural disasters and other emergencies will change the spatial distribution of population at different levels. For example, the COVID-19 pandemic, which erupted globally in 2020, has a rapid spread with a high fatality rate, and the different severities of the pandemic in different countries may lead to a redistribution of the population. While our projection method is a general one, based on the historical population distribution and SSP scenarios, it does not consider such specific impacts yet.

## Usage Notes

Based on the WorldPop dataset, SSPs population projection and other related covariates, we provide a range of future population projections from 2020 to 2100 at a 5-year interval. Each projection product has the spatial distribution of population at an approximately 1 km (30 arc-seconds) spatial resolution. With such a large need for gridded global population projections and to better understand demographic trends, we produce a set of quality projections and make both the code and population projection products available for a wide audience.

To verify the accuracy of the population projection data, we verify the predicted population data at both sub-national and grid levels based on the values of MAE, RMSE and %RMSE. The verification results show that our population projection product has small deviations in most areas of the world and can truly reflect future population changes and distributions.

## Supplementary information

Supplementary Table 1

Supplementary Table 2

Supplementary Table 3

## Data Availability

The global gridded population dataset was created using python 3.9.7 as well as ArcGIS 10.6 software platform, and the code of key steps can be available at Figshare. The code can be downloaded at Figshare (10.6084/m9.figshare.19609356.v3)^46^.
