# Supplementary material for: Projecting 1 km-grid population distributions from 2020 to 2100 globally under shared socioeconomic pathways
Source: Sci Data. 2022 Sep 12;9:563. doi: 10.1038/s41597-022-01675-x (PMC9466344; doi:10.1038/s41597-022-01675-x)
Supplement: Supplementary file 1 — Supplementary Table 1 [file 41597_2022_1675_MOESM1_ESM.docx]

| **Name** | **Resolution** | **Temporal domain** | **Type** | **Source** |
| --- | --- | --- | --- | --- |
| Water Bodies Mask  (Lakes&Reservoirs, Playas, Glaciated Areas) | - | 2018 | Raster | Natural Earth Data (https://www.naturalearthdata.com/downloads/) |
| Global projections of future wilderness | 1 km | 2100 | Raster | Li *et al.* |
| Travel time to cities | ~1 km (30 arc-seconds) | 2015 | Raster | Weiss *et al.* |
| WorldPop | ~1 km (30 arc-seconds) | 2015, 2020 | Raster | https://www.worldpop.org/ |
| WorldPop 3×3,  5×5, 7×7 | ~1km | 2015 | Raster | Calculate from WorldPop 2015 |
| Global Urban Land-Use Change Product (GULCP) | 1 km | 2015,  2020-2100 | Raster | Chen *et al.* |
| Global DEM | 200 m | 2012 | Raster | https://www.nasa.gov/topics/earth/index.html |
| Global Slope | 200m | 2012 | Raster | Calculate from DEM |
| Distance to Road | - | - | Raster | Global Roads Open Access Data Set, Version 1 (gROADSv1):  http://sedac.ciesin.columbia.edu/data/set/groads-global-roads-open-access-v1 |
| Distance to cities (population ≥30,000, ≥50,000, and ≥100,000) | ~1 km (30 arc-seconds) | - | Raster | Calculate based on WorldPop and GULCP |
| Global Land Cover | 5 km | 2015 | Raster | Dynamics of Global Land Cover  (http://data.ess.tsinghua.edu.cn/) |
| SSPs population  projections | Country | 2020–2100 | Continuous | SSP Database (https://tntcat.iiasa.ac.at/SspDb/) |

**Supplementary Table 1. Raster layers used for the global gridded population projection.**
