# Supplementary material for: Projecting 1 km-grid population distributions from 2020 to 2100 globally under shared socioeconomic pathways
Source: Sci Data. 2022 Sep 12;9:563. doi: 10.1038/s41597-022-01675-x (PMC9466344; doi:10.1038/s41597-022-01675-x)
Supplement: Supplementary file 2 — Supplementary Table 2 [file 41597_2022_1675_MOESM2_ESM.docx]

| **Europe (EU)** | | | | | |
| --- | --- | --- | --- | --- | --- |
| **Model** | **MAE** | **RMSE** | **Model** | **MAE** | **RMSE** |
| Sample1 | 10.07 | 125.90 | Sample11 | 9.91 | 103.54 |
| Sample2 | 10.19 | 111.63 | Sample12 | 10.55 | 131.89 |
| Sample3 | 10.44 | 123.42 | Sample13 | 10.42 | 128.82 |
| Sample4 | 9.83 | 107.60 | Sample14 | 10.20 | 121.09 |
| Sample5 | 9.95 | 129.40 | Sample15 | 9.83 | 135.39 |
| Sample6 | 10.47 | 121.69 | Sample16 | 10.21 | 140.97 |
| Sample7 | 9.90 | 110.11 | Sample17 | 10.20 | 154.45 |
| Sample8 | 10.58 | 123.24 | Sample18 | 9.58 | 88.65 |
| Sample9 | 10.48 | 131.16 | Sample19 | 10.72 | 135.63 |
| Sample10 | 10.20 | 135.49 | Sample20 | 10.57 | 140.37 |
| **Latin America (LA)** | | | | | |
| **Model** | **MAE** | **RMSE** | **Model** | **MAE** | **RMSE** |
| Sample1 | 21.59 | 153.11 | Sample11 | 20.91 | 136.13 |
| Sample2 | 21.01 | 147.02 | Sample12 | 21.35 | 142.05 |
| Sample3 | 22.99 | 155.95 | Sample13 | 21.26 | 140.51 |
| Sample4 | 21.35 | 161.10 | Sample14 | 20.65 | 143.41 |
| Sample5 | 20.21 | 143.89 | Sample15 | 21.93 | 147.59 |
| Sample6 | 20.34 | 129.19 | Sample16 | 20.96 | 138.74 |
| Sample7 | 20.97 | 132.43 | Sample17 | 21.10 | 144.90 |
| Sample8 | 20.65 | 153.29 | Sample18 | 21.91 | 172.96 |
| Sample9 | 22.41 | 173.39 | Sample19 | 21.62 | 146.85 |
| Sample10 | 21.01 | 139.28 | Sample20 | 21.09 | 145.93 |
| **Middle East &North Africa (MENA)** | | | | | |
| **Model** | **MAE** | **RMSE** | **Model** | **MAE** | **RMSE** |
| Sample1 | 44.02 | 392.17 | Sample11 | 46.54 | 485.74 |
| Sample2 | 45.13 | 413.74 | Sample12 | 50.40 | 644.02 |
| Sample3 | 49.35 | 511.84 | Sample13 | 49.93 | 490.32 |
| Sample4 | 49.63 | 861.01 | Sample14 | 49.99 | 662.76 |
| Sample5 | 46.04 | 606.10 | Sample15 | 53.80 | 744.90 |
| Sample6 | 52.38 | 714.65 | Sample16 | 52.17 | 750.54 |
| Sample7 | 45.91 | 508.22 | Sample17 | 54.30 | 727.91 |
| Sample8 | 41.94 | 361.15 | Sample18 | 47.90 | 562.41 |
| Sample9 | 46.76 | 538.83 | Sample19 | 52.90 | 714.25 |
| Sample10 | 50.34 | 614.40 | Sample20 | 54.60 | 811.16 |
| **Oceania (OC)** | | | | | |
| **Model** | **MAE** | **RMSE** | **Model** | **MAE** | **RMSE** |
| Sample1 | 3.08 | 16.26 | Sample11 | 2.94 | 15.08 |
| Sample2 | 3.08 | 15.03 | Sample12 | 3.00 | 15.77 |
| Sample3 | 3.13 | 20.19 | Sample13 | 2.98 | 14.54 |
| Sample4 | 2.84 | 12.93 | Sample14 | 3.03 | 16.12 |
| Sample5 | 3.33 | 23.38 | Sample15 | 3.20 | 19.26 |
| Sample6 | 3.05 | 18.20 | Sample16 | 2.78 | 12.89 |
| Sample7 | 2.76 | 13.06 | Sample17 | 2.92 | 15.41 |
| Sample8 | 2.95 | 15.88 | Sample18 | 2.79 | 13.25 |
| Sample9 | 3.17 | 18.40 | Sample19 | 2.87 | 12.75 |
| Sample10 | 2.72 | 13.38 | Sample20 | 3.26 | 19.16 |
| **Russia & the Near Abroad (RNA)** | | | | | |
| **Model** | **MAE** | **RMSE** | **Model** | **MAE** | **RMSE** |
| Sample1 | 5.13 | 33.77 | Sample11 | 5.52 | 41.54 |
| Sample2 | 5.77 | 46.61 | Sample12 | 5.35 | 40.42 |
| Sample3 | 5.84 | 51.83 | Sample13 | 5.61 | 43.05 |
| Sample4 | 5.40 | 41.83 | Sample14 | 5.84 | 47.92 |
| Sample5 | 6.09 | 61.26 | Sample15 | 5.38 | 39.41 |
| Sample6 | 5.88 | 47.24 | Sample16 | 5.05 | 33.53 |
| Sample7 | 5.36 | 38.55 | Sample17 | 5.96 | 50.25 |
| Sample8 | 5.86 | 51.32 | Sample18 | 5.55 | 41.31 |
| Sample9 | 5.36 | 35.79 | Sample19 | 5.70 | 43.58 |
| Sample10 | 5.76 | 50.61 | Sample20 | 5.44 | 36.15 |
| **South &East Asia (SEA)** | | | | | |
| **Model** | **MAE** | **RMSE** | **Model** | **MAE** | **RMSE** |
| Sample1 | 63.92 | 269.78 | Sample11 | 64.30 | 315.54 |
| Sample2 | 65.27 | 353.44 | Sample12 | 65.22 | 333.69 |
| Sample3 | 66.53 | 373.56 | Sample13 | 62.95 | 287.81 |
| Sample4 | 66.08 | 379.87 | Sample14 | 63.65 | 296.77 |
| Sample5 | 64.87 | 296.87 | Sample15 | 65.33 | 315.13 |
| Sample6 | 64.96 | 324.58 | Sample16 | 64.54 | 307.76 |
| Sample7 | 63.63 | 283.01 | Sample17 | 65.07 | 313.94 |
| Sample8 | 64.83 | 326.24 | Sample18 | 64.75 | 305.21 |
| Sample9 | 65.58 | 360.68 | Sample19 | 64.03 | 283.66 |
| Sample10 | 64.33 | 326.95 | Sample20 | 62.12 | 276.45 |
| **Sub-Sahara Africa (SSA)** | | | | | |
| **Model** | **MAE** | **RMSE** | **Model** | **MAE** | **RMSE** |
| Sample1 | 19.96 | 129.96 | Sample11 | 20.05 | 140.24 |
| Sample2 | 19.87 | 113.68 | Sample12 | 22.19 | 165.13 |
| Sample3 | 20.47 | 144.98 | Sample13 | 21.45 | 159.82 |
| Sample4 | 19.76 | 115.39 | Sample14 | 19.49 | 104.03 |
| Sample5 | 20.86 | 155.78 | Sample15 | 22.67 | 199.03 |
| Sample6 | 19.05 | 95.62 | Sample16 | 19.81 | 128.50 |
| Sample7 | 20.00 | 118.07 | Sample17 | 20.24 | 125.90 |
| Sample8 | 21.14 | 153.21 | Sample18 | 20.90 | 135.78 |
| Sample9 | 19.89 | 114.82 | Sample19 | 22.24 | 232.37 |
| Sample10 | 20.51 | 144.68 | Sample20 | 20.60 | 157.91 |
| **United States & Canada (USC)** | | | | | |
| **Model** | **MAE** | **RMSE** | **Model** | **MAE** | **RMSE** |
| Sample1 | 8.33 | 77.40 | Sample11 | 8.08 | 89.13 |
| Sample2 | 7.97 | 91.97 | Sample12 | 8.48 | 104.56 |
| Sample3 | 8.45 | 79.37 | Sample13 | 8.07 | 71.34 |
| Sample4 | 8.99 | 120.03 | Sample14 | 7.81 | 72.31 |
| Sample5 | 8.07 | 77.44 | Sample15 | 8.82 | 116.95 |
| Sample6 | 7.91 | 85.18 | Sample16 | 8.91 | 120.45 |
| Sample7 | 8.49 | 103.34 | Sample17 | 8.32 | 99.64 |
| Sample8 | 8.15 | 58.73 | Sample18 | 7.66 | 79.98 |
| Sample9 | 8.67 | 105.77 | Sample19 | 8.29 | 106.21 |
| Sample10 | 7.71 | 90.22 | Sample20 | 7.72 | 81.82 |

**Supplementary Table 2. Robustness test for sampling method.** We conduct sampling method 20 times for 8 regions. This table shows result of 8 regions.
