# Supplementary material for: Projecting 1 km-grid population distributions from 2020 to 2100 globally under shared socioeconomic pathways
Source: Sci Data. 2022 Sep 12;9:563. doi: 10.1038/s41597-022-01675-x (PMC9466344; doi:10.1038/s41597-022-01675-x)
Supplement: Supplementary file 3 — Supplementary Table 3 [file 41597_2022_1675_MOESM3_ESM.docx]

| **Europe (EU)** | | | | | |
| --- | --- | --- | --- | --- | --- |
| **Country/ Territory** | **Code** | **Population Projection** | **Country/ Territory** | **Code** | **Population Projection** |
| Aland | ALA | RF Model | Isle of Man | IMN | RF Model |
| Albania | ALB | IIASA-WiC POP | Ireland | IRL | IIASA-WiC POP |
| Andorra | AND | RF Model | Iceland | ISL | IIASA-WiC POP |
| Austria | AUT | IIASA-WiC POP | Italy | ITA | IIASA-WiC POP |
| Belgium | BEL | IIASA-WiC POP | Jersey | JEY | RF Model |
| Bulgaria | BGR | IIASA-WiC POP | Kosovo | KOS | RF Model |
| Bosnia and Herzegovina | BIH | IIASA-WiC POP | Liechtenstein | LIE | RF Model |
| Switzerland | CHE | IIASA-WiC POP | Lithuania | LTU | IIASA-WiC POP |
| Cyprus No Mans Area | CNM | RF Model | Luxembourg | LUX | IIASA-WiC POP |
| Northern Cyprus | CYN | RF Model | Latvia | LVA | IIASA-WiC POP |
| Cyprus | CYP | IIASA-WiC POP | Monaco | MCO | RF Model |
| Czechia | CZE | IIASA-WiC POP | Macedonia | MKD | IIASA-WiC POP |
| Germany | DEU | IIASA-WiC POP | Malta | MLT | IIASA-WiC POP |
| Denmark | DNK | IIASA-WiC POP | Montenegro | MNE | IIASA-WiC POP |
| Spain | ESP | IIASA-WiC POP | Netherlands | NLD | IIASA-WiC POP |
| Estonia | EST | IIASA-WiC POP | Norway | NOR | IIASA-WiC POP |
| Finland | FIN | IIASA-WiC POP | Poland | POL | IIASA-WiC POP |
| France | FRA | IIASA-WiC POP | Portugal | PRT | IIASA-WiC POP |
| Faroe Islands | FRO | RF Model | Romania | ROU | IIASA-WiC POP |
| United Kingdom | GBR | IIASA-WiC POP | San Marino | SMR | RF Model |
| Guernsey | GGY | RF Model | Republic of Serbia | SRB | IIASA-WiC POP |
| Gibraltar | GIB | RF Model | Slovakia | SVK | IIASA-WiC POP |
| Greece | GRC | IIASA-WiC POP | Slovenia | SVN | IIASA-WiC POP |
| Croatia | HRV | IIASA-WiC POP | Sweden | SWE | IIASA-WiC POP |
| Hungary | HUN | IIASA-WiC POP | Vatican | VAT | RF Model |
| **Russia & the Near Abroad (RNA)** | | | | | |
| **Country/ Territory** | **Code** | **Population Projection** | **Country/ Territory** | **Code** | **Population Projection** |
| Armenia | ARM | IIASA-WiC POP | Moldova | MDA | IIASA-WiC POP |
| Azerbaijan | AZE | IIASA-WiC POP | Russia | RUS | IIASA-WiC POP |
| Belarus | BLR | IIASA-WiC POP | Tajikistan | TJK | IIASA-WiC POP |
| Georgia | GEO | IIASA-WiC POP | Turkmenistan | TKM | IIASA-WiC POP |
| Kazakhstan | KAZ | IIASA-WiC POP | Ukraine | UKR | IIASA-WiC POP |
| Kyrgyzstan | KGZ | IIASA-WiC POP | Uzbekistan | UZB | IIASA-WiC POP |
| **Latin America (LA)** | | | | | |
| **Country/ Territory** | **Code** | **Population Projection** | **Country/ Territory** | **Code** | **Population Projection** |
| Aruba | ABW | IIASA-WiC POP | Haiti | HTI | IIASA-WiC POP |
| Anguilla | AIA | RF Model | Jamaica | JAM | IIASA-WiC POP |
| Argentina | ARG | IIASA-WiC POP | Saint Kitts and Nevis | KNA | RF Model |
| Antigua and Barbuda | ATG | RF Model | Saint Lucia | LCA | IIASA-WiC POP |
| The Bahamas | BHS | IIASA-WiC POP | Saint Martin | MAF | RF Model |
| Petrel Island | BJN | RF Model | Mexico | MEX | IIASA-WiC POP |
| Saint Barthelemy | BLM | RF Model | Montserrat | MSR | RF Model |
| Belize | BLZ | IIASA-WiC POP | Nicaragua | NIC | IIASA-WiC POP |
| Bolivia | BOL | IIASA-WiC POP | Panama | PAN | IIASA-WiC POP |
| Brazil | BRA | IIASA-WiC POP | Peru | PER | IIASA-WiC POP |
| Barbados | BRB | IIASA-WiC POP | Puerto Rico | PRI | IIASA-WiC POP |
| Chile | CHL | IIASA-WiC POP | Paraguay | PRY | IIASA-WiC POP |
| Colombia | COL | IIASA-WiC POP | Serranilla Bank | SER | RF Model |
| Clipperton Island | CPT | RF Model | South Georgia and the Islands | SGS | RF Model |
| Costa Rica | CRI | IIASA-WiC POP | El Salvador | SLV | IIASA-WiC POP |
| Cuba | CUB | IIASA-WiC POP | Suriname | SUR | IIASA-WiC POP |
| Cura?ao | CUW | RF Model | Sint Maarten | SXM | RF Model |
| Cayman Islands | CYM | RF Model | Turks and Caicos Islands | TCA | RF Model |
| Dominica | DMA | RF Model | Trinidad and Tobago | TTO | IIASA-WiC POP |
| Dominican Republic | DOM | IIASA-WiC POP | United States Minor Outlying Islands | UMI | RF Model |
| Ecuador | ECU | IIASA-WiC POP | Uruguay | URY | IIASA-WiC POP |
| Falkland Islands | FLK | RF Model | Saint Vincent and the Grenadines | VCT | IIASA-WiC POP |
| Grenada | GRD | IIASA-WiC POP | Venezuela | VEN | IIASA-WiC POP |
| Guatemala | GTM | IIASA-WiC POP | British Virgin Islands | VGB | RF Model |
| Guyana | GUY | IIASA-WiC POP | Virgin Islands | VIR | IIASA-WiC POP |
| Honduras | HND | IIASA-WiC POP |  |  |  |
| **United States & Canada (USC)** | | | | | |
| **Country/ Territory** | **Code** | **Population Projection** | **Country/ Territory** | **Code** | **Population Projection** |
| Bermuda | BMU | RF Model | Saint Pierre and Miquelon | SPM | RF Model |
| Canada | CAN | IIASA-WiC POP | United States of America | USA | IIASA-WiC POP |
| Greenland | GRL | RF Model |  |  |  |
| **Middle East &North Africa (MENA)** | | | | | |
| **Country/ Territory** | **Code** | **Population Projection** | **Country/ Territory** | **Code** | **Population Projection** |
| United Arab Emirates | ARE | IIASA-WiC POP | Morocco | MAR | IIASA-WiC POP |
| Bahrain | BHR | IIASA-WiC POP | Oman | OMN | IIASA-WiC POP |
| Algeria | DZA | IIASA-WiC POP | Palestine | PSE | IIASA-WiC POP |
| Egypt | EGY | IIASA-WiC POP | Qatar | QAT | IIASA-WiC POP |
| Iran | IRN | IIASA-WiC POP | Western Sahara | SAH | RF Model |
| Iraq | IRQ | IIASA-WiC POP | Saudi Arabia | SAU | IIASA-WiC POP |
| Israel | ISR | IIASA-WiC POP | Sudan | SDN | IIASA-WiC POP |
| Jordan | JOR | IIASA-WiC POP | Syria | SYR | IIASA-WiC POP |
| Kuwait | KWT | IIASA-WiC POP | Tunisia | TUN | IIASA-WiC POP |
| Lebanon | LBN | IIASA-WiC POP | Turkey | TUR | IIASA-WiC POP |
| Libya | LBY | IIASA-WiC POP | Yemen | YEM | IIASA-WiC POP |
| **South &East Asia (SEA)** | | | | | |
| **Country/ Territory** | **Code** | **Population Projection** | **Country/ Territory** | **Code** | **Population Projection** |
| Afghanistan | AFG | IIASA-WiC POP | Macao S.A.R | MAC | IIASA-WiC POP |
| French Southern and Antarctic Lands | ATF | RF Model | Maldives | MDV | IIASA-WiC POP |
| Bangladesh | BGD | IIASA-WiC POP | Myanmar | MMR | IIASA-WiC POP |
| Brunei | BRN | IIASA-WiC POP | Mongolia | MNG | IIASA-WiC POP |
| Bhutan | BTN | IIASA-WiC POP | Northern Mariana Islands | MNP | RF Model |
| China | CHN | IIASA-WiC POP | Malaysia | MYS | IIASA-WiC POP |
| Christmas Island | CXR | RF Model | Nepal | NPL | IIASA-WiC POP |
| Guam | GUM | IIASA-WiC POP | Pakistan | PAK | IIASA-WiC POP |
| Hong Kong S.A.R. | HKG | IIASA-WiC POP | Spratly Islands | PGA | RF Model |
| Indonesia | IDN | IIASA-WiC POP | Philippines | PHL | IIASA-WiC POP |
| India | IND | IIASA-WiC POP | Palau | PLW | RF Model |
| British Indian Ocean Territory | IOT | RF Model | North Korea | PRK | IIASA-WiC POP |
| Japan | JPN | IIASA-WiC POP | Scarborough Reef | SCR | RF Model |
| Siachen Glacier | KAS | RF Model | Singapore | SGP | IIASA-WiC POP |
| Cambodia | KHM | IIASA-WiC POP | Thailand | THA | IIASA-WiC POP |
| South Korea | KOR | IIASA-WiC POP | East Timor | TLS | IIASA-WiC POP |
| Laos | LAO | IIASA-WiC POP | Taiwan | TWN | RF Model |
| Sri Lanka | LKA | IIASA-WiC POP | Vietnam | VNM | IIASA-WiC POP |
| **Sub-Sahara Africa (SSA)** | | | | | |
| **Country/ Territory** | **Code** | **Population Projection** | **Country/ Territory** | **Code** | **Population Projection** |
| Angola | AGO | IIASA-WiC POP | Mali | MLI | IIASA-WiC POP |
| Burundi | BDI | IIASA-WiC POP | Mozambique | MOZ | IIASA-WiC POP |
| Benin | BEN | IIASA-WiC POP | Mauritania | MRT | IIASA-WiC POP |
| Burkina Faso | BFA | IIASA-WiC POP | Mauritius | MUS | IIASA-WiC POP |
| Botswana | BWA | IIASA-WiC POP | Malawi | MWI | IIASA-WiC POP |
| Central African Republic | CAF | IIASA-WiC POP | Namibia | NAM | IIASA-WiC POP |
| Ivory Coast | CIV | IIASA-WiC POP | Niger | NER | IIASA-WiC POP |
| Cameroon | CMR | IIASA-WiC POP | Nigeria | NGA | IIASA-WiC POP |
| Democratic Republic of the Congo | COD | IIASA-WiC POP | Rwanda | RWA | IIASA-WiC POP |
| Republic of the Congo | COG | IIASA-WiC POP | South Sudan | SDS | RF Model |
| Comoros | COM | IIASA-WiC POP | Senegal | SEN | IIASA-WiC POP |
| Cabo Verde | CPV | IIASA-WiC POP | Saint Helena | SHN | RF Model |
| Djibouti | DJI | IIASA-WiC POP | Sierra Leone | SLE | IIASA-WiC POP |
| Eritrea | ERI | IIASA-WiC POP | Somaliland | SOL | RF Model |
| Ethiopia | ETH | IIASA-WiC POP | Somalia | SOM | IIASA-WiC POP |
| Gabon | GAB | IIASA-WiC POP | S?o Tomé and Principe | STP | IIASA-WiC POP |
| Ghana | GHA | IIASA-WiC POP | eSwatini | SWZ | IIASA-WiC POP |
| Guinea | GIN | IIASA-WiC POP | Seychelles | SYC | RF Model |
| Gambia | GMB | IIASA-WiC POP | Chad | TCD | IIASA-WiC POP |
| Guinea-Bissau | GNB | IIASA-WiC POP | Togo | TGO | IIASA-WiC POP |
| Equatorial Guinea | GNQ | IIASA-WiC POP | United Republic of Tanzania | TZA | IIASA-WiC POP |
| Kenya | KEN | IIASA-WiC POP | Uganda | UGA | IIASA-WiC POP |
| Liberia | LBR | IIASA-WiC POP | South Africa | ZAF | IIASA-WiC POP |
| Lesotho | LSO | IIASA-WiC POP | Zambia | ZMB | IIASA-WiC POP |
| Madagascar | MDG | IIASA-WiC POP | Zimbabwe | ZWE | IIASA-WiC POP |
| **Oceania (OC)** | | | | | |
| **Country/ Territory** | **Code** | **Population Projection** | **Country/ Territory** | **Code** | **Population Projection** |
| American Samoa | ASM | RF Model | Nauru | NRU | RF Model |
| Australia | AUS | IIASA-WiC POP | New Zealand | NZL | IIASA-WiC POP |
| Cook Islands | COK | RF Model | Pitcairn Islands | PCN | RF Model |
| Fiji | FJI | IIASA-WiC POP | Papua New Guinea | PNG | IIASA-WiC POP |
| Federated States of Micronesia | FSM | IIASA-WiC POP | French Polynesia | PYF | IIASA-WiC POP |
| Heard Island and McDonald Islands | HMD | RF Model | Solomon Islands | SLB | IIASA-WiC POP |
| Kiribati | KIR | RF Model | Tonga | TON | IIASA-WiC POP |
| Marshall Islands | MHL | RF Model | Tuvalu | TUV | RF Model |
| New Caledonia | NCL | IIASA-WiC POP | Vanuatu | VUT | IIASA-WiC POP |
| Norfolk Island | NFK | RF Model | Wallis and Futuna | WLF | RF Model |
| Niue | NIU | RF Model | Samoa | WSM | IIASA-WiC POP |

**Supplementary Table 3. Countries and territories of 8 regions.** This table shows the countries and territories of 8 regions and the source of population projection. IIASA-WiC POP indicates that the projection is from SSP scenario, and RF Model indicates the population is projected with RF Model by this research without total constraint.
